# A method for improving the efficiency of DNA extraction from clotted blood samples

**DOI:** 10.1002/jcla.22892

**Published:** 2019-05-10

**Authors:** Maryam Mardan‐Nik, Sara Saffar Soflaei, Atefeh Biabangard‐Zak, Mahla Asghari, Sania Saljoughian, Amir Tajbakhsh, Zahra Meshkat, Gordon A. Ferns, Alireza Pasdar, Majid Ghayour‐Mobarhan

**Affiliations:** ^1^ Cardiovascular Research Center, Faculty of Medicine Mashhad University of Medical Sciences Mashhad Iran; ^2^ Neurogenic Inflammation Research Center Mashhad University of Medical Sciences Mashhad Iran; ^3^ Department of Chemistry, Faculty of Science Ferdowsi University of Mashhad Mashhad Iran; ^4^ Department of Medical Genetics, Faculty of Medicine Mashhad University of Medical Sciences Mashhad Iran; ^5^ Department of Modern Sciences & Technology, Faculty of Medicine Mashhad University of Medical Sciences Mashhad Iran; ^6^ Department of Microbiology and Virology, Antimicrobial Resistance Research Center, Bu Ali Research Center, Faculty of Medicine Mashhad University of Medical Sciences Mashhad Iran; ^7^ Division of Medical Education Brighton & Sussex Medical School Brighton UK; ^8^ Medical Genetics Research Centre, Faculty of Medicine Mashhad University of Medical Sciences Mashhad Iran; ^9^ Division of Applied Medicine, Medical School University of Aberdeen Aberdeen UK; ^10^ Bioinformatics Research Group Mashhad University of Medical Sciences Mashhad Iran; ^11^ Biochemistry of Nutrition Research Center, Faculty of Medicine Mashhad University of Medical Sciences Mashhad Iran

**Keywords:** clotted blood, DNA extraction, frozen, salting‐out

## Abstract

**Background:**

The efficient and rapid extraction of high‐quality genomic DNA from clotted blood samples, which normally have a low yield and poor quality, is an important factor in genomic research. The objective of this study was to develop a simple and safe technique for dispersing the blood clots by the ball bearing metal shots. Normally, such clot samples may not have an acceptable yield by conventional DNA extraction methods. Also, in the present study, we have further investigated to improve salting‐out DNA extraction methods.

**Methods:**

Initially, 500 µL phosphate‐buffered saline (PBS) (1×) and two ball bearing metal shots were added to each tube of the clotted blood sample and then were gently rotated in an electric laboratory rotator for 1 hour at room temperature (18‐25°C). Genomic DNA was then extracted from samples using a modified salting‐out method and a modified QIAamp^®^ DNA Blood Midi Kit and was compared with QIAamp^®^ DNA Blood Midi Kit as a control. An assessment of the concentration and quality of the extracted DNA was performed using the UV‐visible spectrophotometer. The isolated DNA proved amenable to PCR amplification and gel electrophoresis.

**Results:**

The yield and purity of DNA obtained by these three methods were significantly different (*P* < 0.001), with a higher yield in the modified salting‐out method.

**Conclusions:**

Our proposed modified salting‐out method is simple and efficient for the isolation of DNA from old blood clot samples. It is both easy to use and is of low cost in routine laboratory tasks.

## 1 INTRODUCTION

In recent years, molecular techniques have become important tools in identifying populations, finding mutations, and determining the genetic diversity, quantity, and identification of pathogens{Shams, 2011 #2384;Nasiri, 2005 #2385;Nasiri, 2005 #2385}.^1^, ^2^ One of the routine tasks in molecular biology is DNA extraction. Therefore, high‐quantity, as well as high‐quality, DNA extraction is a crucial step in performing further molecular research assays. To choose an efficient extraction method, different influencing factors should be considered. The nature of biological samples and their storage condition, technical issues, time and cost‐effectiveness are some examples.^3^ White blood cells are one of the main sources of DNA genetic analysis which have been used in polymerase chain reactions (PCRs) and other various molecular techniques.^4^, ^5^ DNA extracted from blood samples is crucial for confirmation of genetic abnormalities, as well as application in epigenetic studies and preventive medicine.^1^ The clot remaining from blood samples collected for serum chemistry measurements is also a great DNA source for genetic or forensic studies. Some methods have optimized DNA extraction from clotted blood, which are normally discarded.^8^

All the methods of DNA extraction consist of three basic steps: first, lysis of the cell membrane; second, separation of the DNA from other cellular components especially protein and RNA; and finally, DNA precipitation.^9^

At present, there are many methods for extracting DNA of which phenol‐chloroform extraction, salting‐out procedure, silica‐guanidinium thiocyanate, and commercial kits are commonly used.^10^ However, available commercial kits are usually expensive.^11^ Among manual methods, salting‐out method is a simple, non‐toxic, and inexpensive method for extracting high‐molecular‐weight DNA from peripheral lymphocytes in scientific research.^11^, ^12^ To obtain a favorable DNA yield, optimal concentrations of various salts such as Tris‐HCl, KCl, MgCl2, and NaCl are used as the buffer.^2^, ^12^ Also, recent reports by Xu et al^15^ and Wong et al^16^ have shown that physical breakage of the blood clot into small pieces, before extracting DNA, improves the quality of DNA purification from clotted blood.

Some methods have dispersed blood clots using scalpels^17^ and mesh with centrifuge^15^ after separation in serum‐separator tubes. Crashing of the clot requires using sharp objects which may be hazardous for the personnel.^15^ Other techniques such as using mesh with the centrifuge have limitations such as inaccurate pipetting of blood volume because of clotted blood compared to the obtained cell volume from anticoagulated blood.^15^

By working on a cohort, the Mashhad Stroke and Heart Atherosclerotic Disorders (MASHAD) study,^18^ where we have collected blood samples from participants dating back to more than 7 years ago, along with the fact that genomic DNA extracted from clotted blood samples normally has low yield and poor quality, and we were encouraged to improve the current extraction methods in order to be able to obtain a higher yield. In the present study, we have further developed a simple, safe, and efficient technique for the fragmentation of the clot before DNA extraction processing. We used the ball bearing metal shots as a mixture to maximize the fragmentation of the clot. Also, we have attempted to modify a salting‐out method for DNA extraction with the highest possible yield from blood clots with ethylenediaminetetraacetic acid (EDTA) and tri‐Sodium citrate dehydrate as a blood anticoagulant for both blood homogenization and chelated cations of Mg2^+^ and Ca2^+^ which are necessary when using DNAases. Finally, in this study, the rate of the PCR inhibitor and quality of extracted gDNA were compared between a modified salting‐out method, a modified QIAamp^®^ DNA Blood Midi Kit, and QIAamp^®^ DNA Blood Midi Kit as the control group.

## 2 MATERIALS AND METHODS

### 2.1 Sample collection

We randomly selected thirty‐one clotted blood samples collected as part of the Mashhad Stroke and Heart Atherosclerotic Disorders (MASHAD) study. The MASHAD study is a 10‐year cohort study in an urban population in eastern Iran,^18^ which began in the year 2010. Ten milliliters of peripheral blood was collected in plain tubes. Samples were spun, and the serum was separated. All tubes containing clotted blood were stored at −80°C.

Another set of 32 samples of clotted blood from this cohort were chosen and considered as a comparator group. The study was approved by the Mashhad University of Medical Sciences ethical committee.

### 2.2 Sample preparation

Before proceeding to DNA extraction using a modified salting‐out method^11^, ^19^, ^20^ and modified QIAamp^®^ DNA Blood Midi Kit, 500 µL phosphate‐buffered saline (PBS) (1X) and two ball bearing metal shots, autoclaved in 121°C for 15 minutes, were added to each tube of clotted blood sample and were gently rotated in an electric laboratory rotator for 1 hour at room temperature (18‐25°C). In QIAamp^®^ DNA Blood Midi Kit, blood samples were thawed for 1 hour at room temperature (18‐25°C).

### 2.3 Protocol A: modified salting‐out method

Two milliliters of blood clot was transferred to a tube containing 7 mL of cell lysis buffer solution (CLB) (0320 mmol/L sucrose, 10 mmol/L Tris‐HCl, 2 mmol/L MgCl_2_, 1% Triton X‐100, 4 mmol/L tri‐Sodium citrate dehydrate) (pH 6.50) (Merck, Germany), and the tubes were mixed well by pulse‐vortexing for 2 minutes. After centrifuging at 3800 rpm (1533*g*) for 10 minutes, the supernatant liquid was carefully discarded to waste and 5 mL CLB was added to pellet again, and tubes were well shaken for 2 minutes and centrifuged at 3800 rpm (1533*g*) for 10 minutes. After the supernatant was discarded, 5 mL of low salt buffer containing 10 mmol/L Tris‐HCl, 4 mmol/L MgCl_2_, and 10 mmol/L KCL (known as TKM1) and 0.1 mmol/L Na2EDTA, pH 4.46 (Merck, Germany), was added to the pellet. The samples were mixed for 1 minute and centrifuged at 3800 rpm (1533*g*) for 10 minutes at room temperature. After decanting the supernatant, 1.5 mL of high salt buffer TKM2 containing 10 mmol/L Tris‐HCl, 4 mmol/L MgCl_2_, 8 mmol/L KCL, and 1 mmol/L Na2EDTA, 390 mmol/L NaCl, pH 4.82 (Merck, Germany), along with 100 µL solution of 10% sodium dodecyl sulfate (SDS) (w/v) was added to each tube. The samples were then mixed for 1 minute. This mixture was incubated for 1 hour at 65°C. Then, 500 µL of 6 M NaCl (Merck, Germany) was added. After vigorous shaking for 15 seconds, proteins were removed by centrifugation at 3800 rpm (1533*g*) for 10 minutes. The supernatant liquid was carefully transferred to a new clean tube containing 4 mL cold absolute ethanol (Merck, Germany). The tubes were inverted gently several times, causing long strands of high‐molecular‐weight DNA to appear. The DNA was transferred to a 1.5‐ml sterile microtube along with the addition of cold absolute ethanol. If the DNA cloud were not seen, the solution was transferred to a 1.5‐mL sterile microtube centrifuged at 14000 rpm (20817*g*) for 2 minutes, and the supernatant was then discarded. These steps were repeated several times until the no supernatant solution remained in the tube. The pellet was washed once with 300 μL of 70% ethanol (Merck, Germany) and then centrifuged at 14000  (20817*g*) rpm for 2 minutes. The DNA pellet allowed to be dried for at least 10 minutes at room temperature (18‐25°C) until there was no trace of ethanol. Finally, the DNA was dissolved in 100‐200 μL of the sterile distilled water before storage at −20°C.

### 2.4 **Protocol B: Modified QIAamp** ^®^ **DNA Blood Midi Kit**

Five hundred microlitre phosphate‐buffered saline (PBS) (1X) and two ball bearing metal shots were added to each tube of clotted blood sample. They were then gently rotated for 1 hour. DNA extraction of the clotted blood samples was performed with the QIAamp^®^ DNA Blood Midi Kit (Qiagen, Hilden, Germany) according to the manufacturer's recommendations.

### 2.5 **Protocol C: QIAamp** ^®^ **DNA Blood Midi Kit**

To compare our results, thirty‐two frozen clotted blood samples as the control group were chosen from the MASHAD cohort and thawed at room temperature (18‐25°C) for 1 hour, followed by DNA extraction which was performed using QIAamp^®^ DNA Blood Midi Kit (Qiagen, Hilden, Germany) according to the manufacturer's instructions.

### 2.6 Evaluation of extracted DNA

#### 2.6.1 DNA yield and quality

Two microliters of purified DNA solution was used to measure the quality and quantity of the DNA (ng/μL) (A260/A280) by UV‐visible spectrophotometer (BioTek, USA).

#### 2.6.2 Agarose gel electrophoresis

In order to assess DNA degradation and the molecular weight of the DNA by each method, gel electrophoresis was performed by loading 3 μL of extracted DNA on 0.2% agarose gels (Max Pure, Iran) prepared in 0.5X TBE buffer (Pars tous, Iran).

#### 2.6.3 PCR evaluation of purified DNA

A PCR reaction was performed to check the quality of purified DNA and to determine whether any inhibitory material was interfering with the reaction. For this purpose, exon 3 of the crystalline gamma‐D (*CRYGD*) gene with a fragment of 636 base pairs was amplified in a 10 μL reaction, 3 μL Taq DNA Polymerase 2× Master Mix Red (Ampliqon), one microliter of extracting DNA, and 0.5 pmol forward primer (5′‐TGAATCTCTGTGGGTAATG‐3′) and 0.5 pmol reverse primer (5′ CGTCATTCTGTTGTGAGAACTTCC 3′). The amplification conditions for PCR were as follows: 95°C for 7 minutes to denature the template, followed by 35 cycles of denaturation at 95°C for 30 seconds, annealing at 52°C for 20 seconds, DNA extension at 72°C for 30 seconds, and the final extension at 72°C for 10 minutes. The amplification cycles were performed by PCR system GeneAtlas 322/325 (ASTEC, Seoul, Korea). The amplified DNA was identified by 1.5% agarose gel electrophoresis with DNA Green Viewer™ (Pars tous, Iran) staining and visualized by UV light. Sequencing was performed to confirm the validity of size (639 bp) with gel electrophoresis (Kawsar, Iran).

#### 2.6.4 Real‐time PCR and analysis

The quality of extracted DNA was further evaluated by real‐time PCR. The SNP rs1333049 was amplified with TaqMan^®^ Probes. Amplification was performed in a 12.5 final reaction volume containing 10‐20 ng/µL of DNA and TaqMan^®^ Universal Master with specific primers and probes (L/N: 1706112, Applied Biosystems, Foster City, CA). The PCR conditions were as follows: 95°C for 10 minutes; 45 cycles at 95°C for 15 seconds; and 60°C for 1 minute, using a Roche Thermocycler (LightCycle^®^96, Roche, Germany).

### 2.7 Statistical analysis

Statistical analyses were done using SPSS 17.0 (SPSS Inc, Chicago, Illinois). The assessment of the normality of the data was performed by using the Shapiro‐Wilk test. Non‐normally distributed data are presented as median and percentiles (IQ_1_‐IQ_3_). The Kruskal‐Wallis test was used for comparing non‐normal data among the groups. Statistical significance was set at *P* < 0.05.

## 3 RESULTS

The mean amount of DNA extracted by each method is shown in Table 1. The mean yield and purity of DNA obtained by these three methods were significantly different (*P* < 0.001). A significant difference was also observed in the A260/A280 ratios among those methods (*P* = 0.01). However, when performing the statistical analysis (Mann‐Whitney *U* test for pairwise comparisons), statistically significant differences were not found for either of the gDNA yield (*P* = 0.4) or the A260/A280 ratios (*P* = 0.06) between the modified salting‐out method and modified QIAamp^®^ DNA Blood Midi Kit method.

**Table 1:** Yield and A260/A280 ratio of DNA purified from clotted blood

Genomic DNA extraction method	Number	Genomic DNA yield (ng/µL)			A260/A280 ratio		
		Min	Max	Median (Q1‐Q3)	Min	Max	Median (Q1‐Q3)
Modified salting‐out method^*^	31	15.53	244.48	66.51 (44.93‐87.54)	1.4	1.8	1.7 (1.6‐1.7)
Modified QIAamp^®^ DNA Blood Midi Kit	31	11.74	260.25	78.12 (43.11‐134.70)	1.5	1.8	1.7 (1.7‐1.8)
QIAamp^®^ DNA Blood Midi Kit	32	8.40	108.66	38.13 (20.28‐58.23)	1.2	2.8	1.7 (1.6‐1.7)
*P*‐value	<0.001				0.01		

Values are expressed median (Q1‐Q3). Comparisons were performed by Kruskal‐Wallis test.

**Figure 1:**
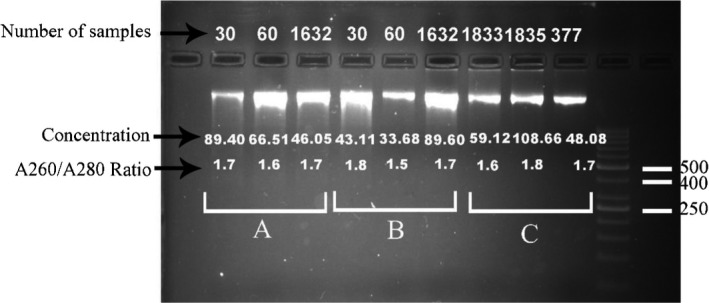
Comparison of genomic DNA purified from clotted blood by each extraction method on 2% agarose gel. Stained with DNA Green Viewer™. A: modified salting‐out method, B: modified QIAamp^®^ DNA Blood Midi Kit, C: QIAamp^®^ DNA Blood Midi Kit. Lane 11:50‐bp DNA ladder (Fermentas)

**Figure 2:**
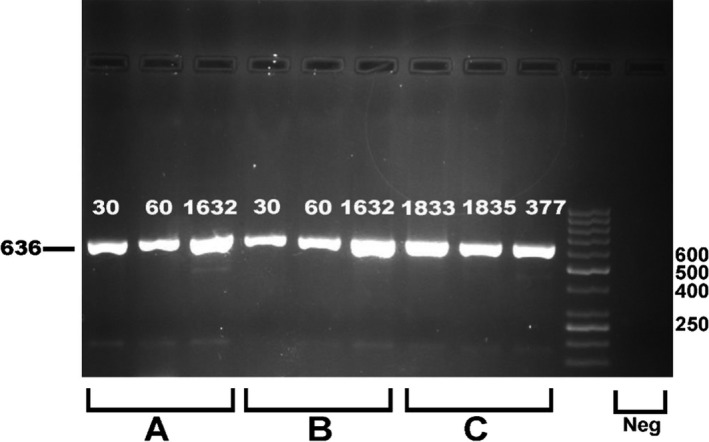
Comparison of PCR products obtained with DNA purified blood by each extraction method with primers of CRYGD gene. Electrophoresed on 1.5% agarose. A: modified salting‐out method, B: modified QIAamp^®^ DNA Blood Midi Kit, C: QIAamp^®^ DNA Blood Midi Kit. Lane 10:50‐bp DNA ladder (Fermentas); Lane 11: negative control

The quality of the DNA was fairly uniform in both modified salting‐out and modified QIAamp^®^ DNA Blood Midi Kit methods. It was then compared with the extracted DNA samples using QIAamp^®^ DNA Blood Midi Kit as the control group (Figure 1). Comparisons of the quality of genomic DNA (gDNA) purification in DNA extraction methods with and without physical breakage of blood clots are presented in Table 3. The analysis of PCR products (Figure 2) and real‐time PCR showed that DNA purified from clotted blood samples could be used to amplify and genotype the exon 3 of the crystalline gamma‐D (*CRYGD*) gene and SNP rs1333049 successfully. Cost comparisons for different gDNA extraction methods are shown in Table 2.

**Table 2:** Time and cost comparison for different genomic DNA extraction methods

Genomic DNA extraction method	Cost estimate per sample	Cost of gDNA^a^ extraction per 100 samples	Process duration for per sample
Modified salting‐out method	≈20¢	≈20¢$	≈3 h
Modified QIAamp^®^ DNA Blood Midi Kit	<9$	$887.00	≈1 h:45 min
QIAamp^®^ DNA Blood Midi Kit	<9$	$887.00	≈1 h:50 min

^a^ Based on information from the company website accessed in July 2017.

**Table 3:** Comparing the quality of genomic DNA (gDNA) purification in DNA extraction methods with and without physical breakage of blood clots

		Volume blood clots (mL)	Process duration per sample	Mean DNA yield (μg)*	Mean DNA concentration (ng/µL)**	Mean OD260/OD280 ratio	References
Extraction method with the physical fragmentation of clot	Sterile scalpel with salting‐out method	7	N/A	367	52.42	1.84	1
	20‐gauge wire mesh by centrifugation with organic solvent^a^	4	N/A	36.6	9.15	N/A	2
	Copper mesh (pore size, 250 µm) by centrifugation with kit	0.3	N/A	19.02	63.4	1.80	3
Extraction method without physical fragmentation of clot	Salting‐out method	0.3	≈45′	7.9	26.33	1.73	4
	Modified salting‐out method	5	≈1h	40.47	8.09	1.75	5

N/A, not applicable.

a After erythrocyte lysis and proteinase‐K digestion of the fragmented clot, DNA was precipitated with isopropanol in the presence of glycogen.

* Represents DNA concentrations reported in some publications in which DNA is extracted from blood clot which has been stored from few months to several years, whereas some studies have used fresh whole blood.

** We calculated based on available information.

## 4 DISCUSSION

In previous studies, the physical breakage of dispersing blood clots was carried out by different methods. The sterile scalpel and mesh were used for a quick isolation of DNA from clotted blood samples.^15^, ^16^, ^21^, ^22^

This study developed an alternative method to process a large number of blood clots for isolation of high‐quality DNA, which involves the use of the ball bearing metal shots to homogenize and break down the blood clot, followed by adding phosphate‐buffered saline (PBS) to maximize the volume of blood in liquid form. It is believed that PBS will increase the buffy coat volume.^23^

Our results showed a significant increase of DNA extraction in modified salting‐out method and modified QIAamp® DNA Blood Midi Kit when compared with QIAamp^®^ DNA Blood Midi Kit (*P* < 0.001, 75.43 ± 47.12 and 91.48 ± 63.07 vs 42.46 ± 26.21), suggesting that the quantity of DNA was higher when a mechanical device was used. This observation is consistent with previous results which indicated a mechanical breakage of the clots is useful for enhancing the amount of DNA.^8^, ^15^

Therefore, it seems that the use of ball bearing metal shots in blood clot containing tubes is a simple and safe mechanical device to break down the blood clot. The superiority of such method can be listed as follows: (a) the handling of the clotted sample is minimized; (b) reduced risk of possible external contamination of the samples; (c) improving the DNA yield through the efficient disintegration of the clot; (d) speeding up the extraction procedure as blood clots may hinder the conventional extraction procedures; and (e) improving the safety as there are no sharp objects used and human intervention as well as exposure to hazardous materials is minimized.

Regarding evaluation of the extracted DNA, Figure 2 indicates that the extracted DNA was successfully amplified via PCR genotyping. The quantification of gDNA was more accurately determined using real‐time PCR. We did not identify prominent inhibitory factors in the solution. These findings demonstrated the quality and quantity of DNA samples purified from clotted blood, and this method would also help to use frozen blood clots as a source of DNA in many areas of molecular biology.

In practice, the use of the ball bearing metal shots to homogenize and disperse the blood clot followed by a routine method for extraction of DNA is simple and safe. The advantage of the modified salting‐out method is simple and inexpensive (Table 2). The main challenge in this method is pipetting in each step to dissolve the pellet. The disadvantages of QIAamp^®^ DNA Blood Midi Kit include needing to use a fixed‐angle rotor centrifuge as well as expensive reagents. Modified QIAamp^®^ DNA Blood Midi Kit is a suitable choice for enhancement of the speed of DNA purification and minimizes the possibility of cross‐contamination. The results of using both physical and chemical methods for the extraction of DNA from the old blood clotted samples are compared with the results of using only the chemical methods in Table 3. It shows the reduction of time required for DNA extraction, and improvement of the quality and quantity of DNA is fairly reduced.

In conclusion, the results showed that this method is simple, safe, and more economical for isolation of DNA from frozen blood clots. Although this method is beneficial and practical for either fresh or clotted blood, the ball bearing metal shots for the fresh blood, which is a homogeneous suspension, may be skipped.

## CONFLICT OF INTEREST

The authors have no conflict of interest to declare.
